# Utilization of a Mobile Dental Vehicle for Oral Healthcare in Rural Areas

**DOI:** 10.3390/ijerph16071234

**Published:** 2019-04-07

**Authors:** Sherry Shiqian Gao, Madeline Jun Yu Yon, Kitty Jieyi Chen, Duangporn Duangthip, Edward Chin Man Lo, Chun Hung Chu

**Affiliations:** Faculty of Dentistry, The University of Hong Kong, Hong Kong 999077, China; yonjunyu@hku.hk (M.J.Y.Y.); kjychen@hku.hk (K.J.C.); dduang@hku.hk (D.D.); hrdplcm@hku.hk (E.C.M.L.); chchu@hku.hk (C.H.C.)

**Keywords:** mobile dental vehicle, oral healthcare, rural area, inequality

## Abstract

Oral diseases remain one of the major global public health challenges, and the worldwide urban–rural disparities in oral health are significant. Residents in rural areas generally suffer from a higher prevalence and severity of dental caries and periodontal disease, yet they face numerous difficulties and barriers in accessing oral healthcare. Conventional strategies, such as building of dental clinics or, hospitals, or the provision of outreach services by using disposable materials, are neither practical nor effective in rural settings. Mobile dental vehicles (MDVs) have been proposed as an alternative strategy to supplement the traditional oral healthcare in many regions. They have usually been utilized in school-based oral health programs, providing dental care to the homeless or migrants, and screening programs for the population for various oral diseases. Due to their high mobility, MDVs are particularly valuable for the underserved populations living in rural areas. The advance of dental devices enables MDVs to be operated in a self-sufficient manner. This allows the MDV to function almost as well as a conventional dental clinic, providing a variety of dental treatments, including scaling, restoration, and oral surgery. This article discusses the use of MDVs as a solution to urban–rural inequality in receiving oral healthcare.

## 1. Introduction

Although both governmental and non-governmental organizations have made great efforts to promote oral health worldwide, the oral health situation has not improved in the last few decades. According to the Global Burden of Disease (GBD) study published in 2015, oral diseases remain one of the major global public health challenges [1]. Nearly half of the global population suffered a certain level of disability from undesirable oral conditions. The total disability-adjusted life years due to oral conditions (tooth loss, severe chronic periodontitis, untreated dental caries, etc.) was 16.9 million in 2015, and has increased by 64% since 1996 [1]. Untreated dental caries in permanent teeth affected more than 2.5 billion people worldwide, which was the most prevalent oral disease listed in the GBD 2015 [1]. Dental caries is an ambulatory care sensitive condition. Emergency admission of people suffering from dental caries and its complications is very common in rural areas, which indicates the poor overall quality of primary and community oral healthcare received by people in rural areas [2].

## 2. Urban–Rural Disparity in Oral Health Conditions

Studies have reported on the urban–rural disparities of different oral health-related conditions [3,4,5]. People living in rural areas are more likely to suffer dental caries and have untreated decay. Preschool children living in rural areas showed a higher prevalence of dental caries than urban children [3,5]. A similar result was also observed in older people [6]. In addition, some studies supported the idea that rural residents presented a worse periodontal health status than urban residents [3,6,7]. The prevalence of gingivitis and calculus in 12-year-old children was higher if they were living in rural areas [6]. Fewer middle-aged (35- to 44-year-old) people in rural areas were classified as healthy using the community periodontal index [3]. Furthermore, significantly more people living in rural areas reported low oral health-related quality of life than in urban areas, suggesting that poor oral conditions have a greater negative impact on rural inhabitants [8].

The rationales behind these disparities have been under discussion. The inequalities of oral health services, accessibility, utilization, oral health knowledge and practices, and health insurance coverage might exist between urban and rural areas [4]. It has been well-accepted that people’s dental-related behaviors (toothbrushing, smoking and drinking habits, frequency of dental checkups, etc.) and socioeconomic background (household income, education level, etc.) are related to their oral health [9]. These factors can be considered as the mediators between the geographic living areas and oral health conditions. People living in rural or remote areas are often impoverished, with a low income, low education level, unfavorable living environment, and limited access to healthcare [4]. Therefore, their knowledge, attitude, and behaviors regarding oral health practice are generally inadequate and insufficient [10].

## 3. Urban–Rural Inequalities in Oral Healthcare

The urban–rural inequality in oral healthcare remains prevalent worldwide. Significant difficulties regarding the availability, accessibility, and affordability of standard oral healthcare have been observed in rural areas [11]. These difficulties can be illustrated by a shortage of dental personnel, low population density and geographic isolation, lack of electricity and water supply, limited economic resources, and an aged rural population.

There is often a shortage of healthcare personnel in rural areas [12]. Clinics in rural areas consistently face difficulties in recruiting and retaining dentists and paradental staff as the patient pool is small over a vast area and the job opportunities or prospects in rural areas are not considered to be as favorable as in busy urban areas [12].

Rural areas are almost always sparsely populated. Due to the low population density and geographical isolation, healthcare facilities are not common or may not be situated in close proximity to most of the rural residents, and regular oral health services become out of reach [13]. Even when these residents are willing to travel to the nearest dental facility, public transportation systems are often limited or even non-existent for rural areas, and road conditions may be unfavorable for long-distance travel [14]. For these reasons, rural residents, particularly those with low income, face difficulties travelling to seek oral healthcare from the nearest clinic or medical center, which may be located far away in the city.

In rural areas, the electricity and water supply is often insufficient or unstable [15]. Therefore, it may be impossible to set up a fixed site dental clinic with, for example, powered dental equipment and radiography units in rural areas, which limits the treatment choices available and dental conditions that can be properly addressed.

Rural populations have limited economic resources. They presented a lower average family income than people living in urban areas, and their priority for oral health maintenance may be very low. Therefore, they may not have the ability or incentive to afford conventional oral healthcare [4].

Rural areas tend to have a greater proportion of older people as the younger generation prefer to seek education and working opportunities in big cities [16]. This group of older people are less able to access oral healthcare due to their physical health condition, and they may be ill-informed about dental healthcare services compared to their counterparts in the cities where older people may be systematically taken care of in elderly homes and institutions.

Strategies have been proposed to address the urban–rural inequalities in oral healthcare. Each of these strategies can solve some of the aforementioned barriers, but none are able to be perfectly adopted in rural areas (Table 1). It would be ideal if well-equipped dental clinics could be set up in rural areas. However, the power and water supply may be unstable or non-existent and, even so, the start-up cost of dental clinics is very high and it might be challenging to recruit dental personnel to these rural clinics. In addition, due to the low population density, the utilization of the clinic is often low and, thus, the cost-effectiveness is also very low.

Inviting rural residents to hospitals in urban areas may be another solution to meet their dental needs without building a local clinic. Oral health providers or organizations can schedule shuttle buses between villages and cities and allocate treatment time slots for these patients in dental hospitals. However, because of the sparse population density in rural areas, running shuttle buses at a regular frequency through the vast landscape might not be cost-effective and dental resources may be thinned out. This strategy may be useful for population-based dental examination and screening but may not be a practical option to deliver timely dental treatment to this group of people. In addition, people who have difficulty moving or are not suitable for long travel will be left underserved.

Another suggestion is for some organizations to provide outreach services to rural people by using disposable dental equipment and materials. This strategy has high mobility and is relatively low-cost. However, due to limitations regarding equipment, certain procedures such as radiography, suction, and moisture control may not be available. Therefore, only simple treatments like fluoride therapy, atraumatic restorative treatments, or simple scaling can be provided to patients. In addition, inadequate infection control of the equipment and environment may increase the risk of virus transmission [17].

Since all the above mentioned strategies have significant limitations, they are not ideal for delivering regular oral healthcare to people living in rural areas.

## 4. Use of Mobile Dental Vehicles in Oral Healthcare

Mobile dental vehicle (MDVs) can be adopted to address the oral healthcare needs of different populations, such as through school-based oral health programs, and the provision of oral care service to the homeless, people who are temporarily displaced, migrants, people living in rural or remote areas, people from low socioeconomic communities, etc. [18].

**Figure 1:**
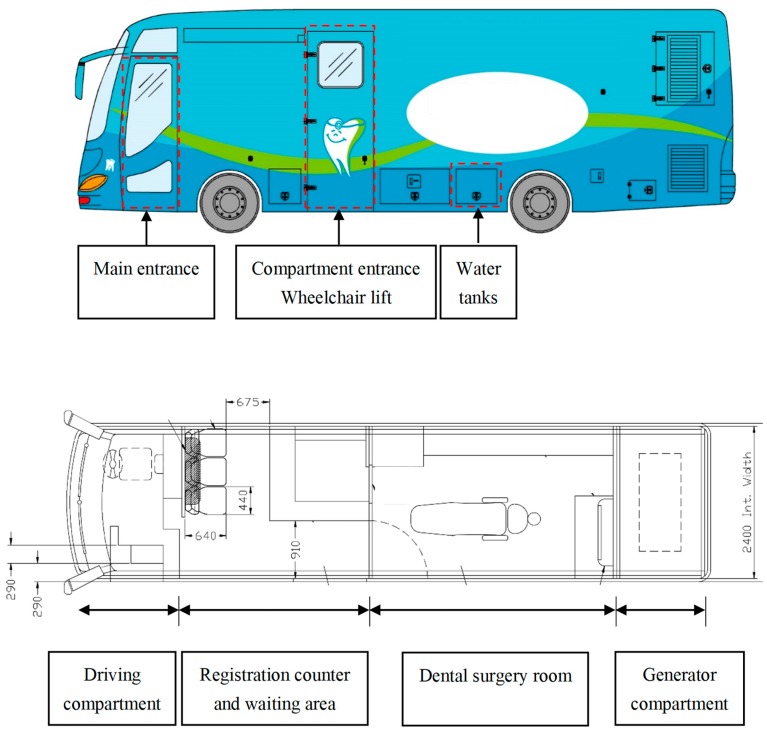
A typical mobile dental vehicle.

An MDV can be a truck or a bus. The layout of a typical MDV is shown in Figure 1. This MDV was renovated from a single-deck bus. It consists of four parts: a generator compartment, a driving compartment, a registration counter and waiting area, and a dental surgery room. The MDV is equipped with a generator set providing a three-phase power supply, which can support the electricity needed by the lighting and dental clinic. The MDV has three water tanks: a fresh water tank, a drain water tank, and a recycle water tank. The fresh water tank contains fresh water for clinical use. The drain water tank is used to store waste water before it is safely disposed of. The recycle water tank continuously circulates, generating a moving current that can provide the vacuum environment needed to create a suction force for the clinical aspirator.

The driving compartment is in the front of the bus. The driver of the MDV has to be trained or may be required to pass a specific driving license examination for such special purpose vehicles in some regions. The registration counter is behind the driving compartment. The seats in waiting area are equipped with seat belts which can be used by the dental team during transportation. Computer sets are usually available in the MDV for patient registration and treatment records. The use of an electronic health record sharing system can enhance the accuracy and efficiency of obtaining patients’ records. It also provides dentists with essential and updated medical and dental histories through an information infrastructure within both the public and private healthcare sectors.

**Figure 2:**
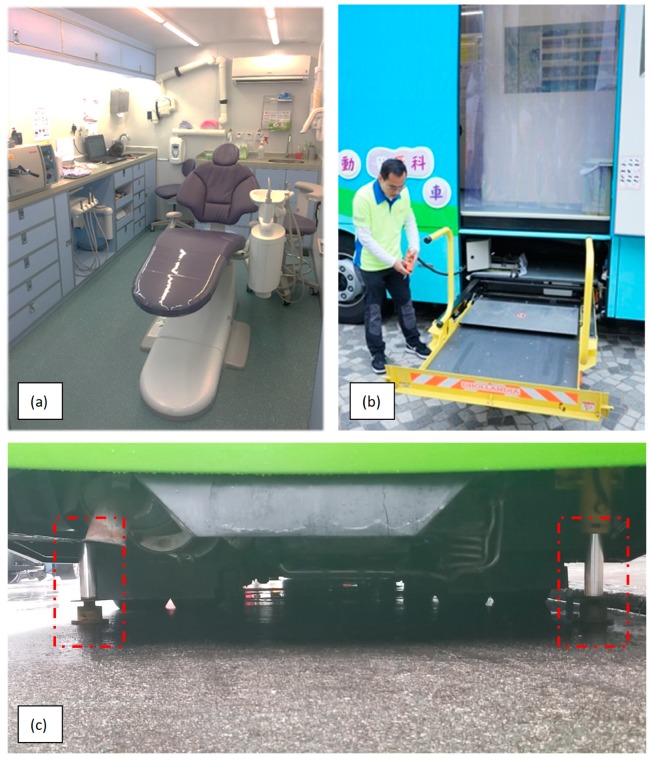
The dental surgery room (**a**), a wheelchair lift (**b**), and stabilizers (**c**) of a mobile dental vehicle (in the red square).

The surgery room is the highlight of the MDV, with a fully-equipped dental unit supplied with equipment and instruments that are available in most dental clinics (Figure 2a). The dental chair is a standard type for supine and upright positions. High-power suction is available in the dental unit and permits treatments requiring large volumes of lavage and improves infection control. There are movable chairs for both operator and assistant. Radiographic equipment for intraoral films is available, with a digital complementary metal oxide semiconductor sensor that allows instant viewing and storage of radiographic records with the reader and computer, forgoing the need for film processing solutions, i.e., developer and fixer solutions. The door of the surgery room is paneled with lead lining for radiation protection. The design of the dental surgery compartment should ensure adherence to the “As Low As Diagnostically Acceptable” principle in relation to the exposure of patients to radiation, and to minimize the occupational health risks of dental healthcare workers. Drawers in the benches store most of the common dental equipment needed. These are installed with latches to prevent drawers from sliding out when the van is in motion or parked on a slope. A bench-top sterilizer, typically an autoclave, is also installed for sterilization purposes, allowing a wider range of instruments to be used on the MDV.

Additional facilities are customized for the MDV for better accessibility. There is a wheelchair lift connected to the compartment entrance (Figure 2b). The surgery room and entrance are also designed to accommodate patients in wheelchairs so that the handicapped may also access MDV services. There are four stabilizers attached to four wheels of the bus (Figure 2c). They can stabilize and keep the MDV horizontal even when the vehicle is parked on a slope. The interior of the MDV is air-conditioned to control the temperature and humidity of the operating environment. To further advance infection control in the limited working space, air filtration is essential for providing clean fresh air and to circulate the air to avoid contamination of wounds. A laminar flow technique can be employed to provide unidirectional or uniform air flow and prevent turbulences. The instrument tables and the operation field itself must be in the zone where filtered air is first introduced. The arrangement of different working places in an operating MDV is challenging because of the limited space. Nevertheless, hygiene considerations must be introduced in the MDV to minimize the risk of cross-infection.

The advance of dental devices enables MDVs to be operated in a self-sufficient manner. They can function as a convention dental clinic that provides a variety of dental treatments, such as scaling, restoration, and oral surgery for a large number of patients in a controlled environment with a high standard of infection control.

## 5. Use of a Mobile Dental Vehicle to Deliver Oral Healthcare to Rural Areas

As a self-sufficient system to deliver dental care, an MDV is able to overcome the major barriers facing the delivery of oral healthcare to rural areas, as mentioned in the previous section (Table 1). The MDV service does not require that dental professionals stay in the rural area all the time. Dentists based in urban clinics and hospitals can serve in an MDV part-time, in particular, those dentists who are under advanced training in community dentistry. This arrangement will bring in professional service and advice within a regular timeframe for the underserved population, addressing the shortage of manpower in the rural areas. The high mobility of an MDV allows dental services to be provided in isolated locations where public transport connections are limited. Multiple visits to different sites and different routings can be arranged periodically, which is particularly valuable for service delivery in sparsely populated areas. An MDV is always equipped with a generator set to provide stable electricity and tanks for storing clean water so that it can continue to provide advanced dental treatments regardless of the power and water supply. The wheelchair-friendly design makes it possible for MDVs to serve those with disabilities. People who are ill-suited for lengthy commutes can also receive dental care from an MDV parked near to their area of residence.

An MDV also has limitations when being adopted for oral health service to rural areas. First, MDVs require a relatively high cost of maintenance. A specialized technical team is needed to run and maintain the vehicle. The team would need to possess knowledge of vehicle maintenance and building facilities, as well as the mechanics, hydraulics, electrics, and electronics, in order for every outreach to run smoothly. Second, MDVs have limited space to care for patients with special needs, who require the more advanced and complex supporting facilities that are normally only available in hospital surgery units. Third, geography and weather-related hazards can affect the availability of MDV service. Accessibility to rural locations depends very much on the topography of the area and road conditions, which may be undermaintained. To complicate the situation, unforeseen weather conditions may block off sections of roads altogether. Although an MDV service has its limitations for delivering total dental care service to rural areas, these limitations are normally only confined to operation frequency and treatment difficulty. To have a dental clinic on four wheels is the major advantage of an MDV that makes it the most promising strategy to deliver oral healthcare to rural populations.

## 6. Other Benefits of Using a Mobile Dental Vehicle

In addition to the aforementioned characteristics and benefits, there are other advantages in running MDV dental care services in rural areas. Compared to a fixed dental clinic, a moderate start-up cost is required for running an MDV service. One study reported that establishing an MDV only costs about 200,000 to 300,000 USD, which is much less than what is needed to set up a conventional dental clinic [19].

Moreover, studies reported that utilization of an MDV is a cost-efficient strategy, especially when expanding the size of service recipients [20,21]. A study was conducted in Thailand comparing the unit costs of dental treatments between the community-based mobile dental clinic and fixed site dental clinic. The results showed that various services can be provided in the community-based mobile dental clinic; the unit cost of treatments in the community-based mobile dental clinic was lower than that of the same treatments provided in the hospital dental clinic [22]. Therefore, the cost-efficiency of an MDV service is particularly beneficial to poor people that cannot afford to seek treatment in conventional dental clinics.

Apart from benefiting the rural population, an MDV program can be a good opportunity to educate dental professionals. A program named “Oral Health on Wheels” provided a mobile dental hygiene clinic to the underserved population [23], and dental hygiene students that joined were surveyed for their perspectives of this program. Students provided positive feedback that the MDV program helped them to understand the dental care needs of the underserved population and enhance their confidence in clinical skills. An MDV service to rural areas may bring similar benefits for the participating dental professionals. Most dentists choose to work in urban areas, and their understanding of the dental care needs in rural areas can be very limited. Dental professionals can work voluntarily or as part-time staff to serve the people living in rural areas. An MDV program can become a channel between dental professionals and rural people, providing dental professionals with a convenient and unique opportunity to engage in community service and serve the public in this special environment without imposing too much constraints on the resource-poor rural areas. Through serving the rural population, dental professionals can visualize and better understand the oral health situations in rural areas and develop empathy for this section of the population that have great oral health needs. Hence, dentists have better engagement with the community in rural areas through their MDV dental service. An MDV program can be a bidirectional win–win strategy that benefits both the people living in rural areas and the participating dental staff.

## 7. Conclusions

There are significant oral health inequalities between people living in urban and rural areas. With high mobility, self-sufficiency, and cost-effectiveness, the use of MDVs can be a promising strategy to deliver oral healthcare to rural populations.

## Figures and Tables

**Table 1:** Existing strategies to provide oral healthcare in rural areas.

		Fixed Dental Clinic	Invite People to Clinic in Urban Area	Outreach Service	Mobile Dental Van
Overcome the barriers of oral healthcare in rural areas (Yes/No)	Shortage of dental personnel	No	Yes	Yes	Yes
	Low population density	No	Yes	Yes	Yes
	Geographic isolation	Yes	Yes	Yes	Yes
	Lack of electricity and water supply	No	Yes	No	Yes
	Limited economic resources	No	No	Yes	Yes
	Aged rural population	Yes	No	Yes	Yes
Other drawbacks of each strategy		High start-up cost; low cost-effectiveness.	Low cost-effectiveness; only for those fit for long-distance travel.	Unsatisfactory suction and moisture control; limited treatment variety; risk of cross-infection.	Relatively high ongoing cost; limited service to special care patients; weather-related problems.
